# Missing assent: a global systematic review of ethical practices in HIV research with men who have sex with men (MSM) under 18

**DOI:** 10.1186/s12910-026-01378-7

**Published:** 2026-02-03

**Authors:** Worawalan Waratworawan, T Charles Witzel, Sin How Lim, Thomas E. Guadamuz

**Affiliations:** 1 Department of Society and Health, Faculty of Social Sciences and Humanities, Mahidol University, Nakhon Pathom, Thailand; 2 Mahidol Center for Health, Behavior and Society, Faculty of Tropical Medicine, Mahidol University, Bangkok, Thailand; 3 Institute for Global Health, University College London, London, UK; 4 Department of Social and Preventive Medicine, Faculty of Medicine, University of Malaya, Kuala Lumpur, Malaysia

**Keywords:** HIV research, MSM, Minor, Informed consent, Informed assent

## Abstract

**Background:**

Ethical safeguards are critical in HIV research involving minors, especially vulnerable groups such as men who have sex with men (MSM) under 18 years. This systematic review explores how informed assent and consent practices are reported, guided by the 2016 Council for International Organizations of Medical Sciences (CIOMS) guidelines.

**Methods:**

A systematic review was conducted of peer-reviewed studies from 2010 to 2023 involving MSM under 18 years, using PubMed, Embase, and Scopus databases. Key ethical components were extracted and synthesized thematically.

**Results:**

Of 410 articles screened, 65 met inclusion criteria. All included studies reported informed consent, but only 20% (*n* = 13) included informed assent. Most were conducted in high-income countries (64%), with no representation from low-income settings. Only 7% involved minors living with HIV. Quantitative methods were predominant (82%), and 20% used online platforms. Waivers of parental permission were reported in 27% of studies, while only 3% obtained parental permission. Four studies described tailored consent/assent approaches, such as simplified language, comprehension checks, and involvement of minor advocates.

**Conclusion:**

This review highlights inconsistencies in how informed assent is addressed in HIV research involving MSM minors. Only one in five studies described ethically appropriate, youth-tailored processes. Limited use of waivers and adapted assent procedures points to ongoing gaps in aligning research with ethical standards. Future studies should adopt clearer, age-appropriate consent practices, ensure transparent reporting, and strengthen researcher training in ethics involving vulnerable minors.

## Introduction

 Despite progress in HIV treatment and prevention, the scale of the epidemic remained alarming. In 2023, approximately 9.3 million people were still living with HIV without access to treatment. Globally, around 630,000 people died of AIDS-related illnesses in that year alone, including 76,000 children under the age of 15, accounting for one in every eight AIDS-related deaths. HIV prevention efforts showed uneven progress. Key populations, such as men who have sex with men, transgender individuals, people who inject drugs, as well as youth, remained underserved in access to effective prevention services [1].

Given these ongoing challenges and disparities, HIV research continued to receive substantial funding support [2–6], recognizing that the epidemic required multidisciplinary responses and cross-sector collaboration. A large number of HIV studies were conducted using both observational and interventional methods [7, 8], exploring diverse areas such as HIV prevention [8, 9], HIV testing [10–13], care, and retention [14]. Much HIV research focused on key populations, including men who have sex with men (MSM), transgender women, people who use drugs, people in prisons and other closed settings [15–20], as well as migrant workers [21–23], people from minority ethnic backgrounds [24–26], and young people [27, 28].

Adolescents became a key target population in HIV interventions, particularly young MSM, who experienced overlapping vulnerabilities due to both their age and sexual orientation. Indeed, young MSM often faced complex social contexts, including stigma and discrimination, lack of family support, limited access to LGBTQ+-friendly healthcare services, and insufficient knowledge and skills related to HIV prevention. These challenges were compounded in countries where same-sex relationships were criminalized. For instance, as of 2022, 31 out of 54 African countries, including Nigeria, Uganda, and Malawi, still enforced laws against consensual same-sex acts, which further restricted access to HIV prevention and care services [29]. Such legal barriers contributed to broader obstacles in accessing preventive measures such as HIV pre-exposure prophylaxis (PrEP) and regular HIV testing. These challenges may be further amplified in low-income countries, where factors such as sociocultural stigma [30, 31], criminalization of same-sex relationships [29], and limited research infrastructure present substantial barriers to conducting ethical research involving MSM minors. The criminalization of homosexuality in many low-income settings [29] can heighten risks for both participants and researchers, potentially discouraging ethical review boards or institutions from approving such research. Moreover, restrictive legal environments may prohibit the enrollment of sexual minority minors without parental permission, even in contexts where such disclosure could place youth at risk. These factors may partly explain the absence of studies involving MSM minors in low-income settings. Within this context, research involving vulnerable populations such as young MSM had to prioritize ethical considerations, particularly with regard to protecting participants’ rights, safety, and well-being, which are core principles essential to conducting research responsibly and with integrity [30–32].

All ethically conducted research needed to protect participants from harm and had to outline how participants’ information would be safeguarded from improper use. In HIV research, this process had to be conducted with considerable sensitivity, since it dealt with an issue linked with experiences of stigma, discrimination, and potential mental health impacts, especially among MSM minors [33–36]. Therefore, the recruitment and enrollment process for research participants was critical, as these processes required a careful balance of risks and benefits, as well as the use of accessible language when describing project details. In many settings, researchers also had to ensure that their participants were of legal age to participate, especially MSM minors. Furthermore, when conducting research with MSM minors, participants needed to be given sufficient time to decide whether or not to take part. Sometimes, it was necessary to obtain parental consent, a waiver of parental permission, or even a proxy. Various consent options could be applied when conducting research with MSM minors, including parental or guardian consent, informed assent with parental permission, waivers, and proxy permission. Each of these approaches had different ethical and legal considerations, which had to be carefully evaluated based on the study context and jurisdiction (Table 1). These practices demonstrated the researchers’ care for the participants’ safety, while also ensuring that minors were protected and cared for while engaging in the study process without coercion.

**Table 1:** Types of assent/consent [38–42]

Type of Assent/Consent	Definition	Strengths	Challenges
Parental/guardian permission	Requires explicit permission from a parent or legal guardian before a minor can participate	Ensures legal and ethical compliance; protects minors from potential risks	May discourage participation due to stigma, confidentiality concerns, or lack of parental/guardian support
Informed Assent + Parental permission	The minor agrees to participate (assent), but legal authorization is still required from a parent/guardian	Gives minors a voice in decision-making; maintains legal protection	Similar challenges as parental/guardian permission, including potential reluctance to disclose sensitive topics such as sexual identity, sexual activities, violence and substance use
Informed Assent + Proxy Permission	The minor agrees to participate (assent), and designated individual (not a parent/legal guardian) is authorized to provide permission on behalf of the minor	Provides an alternative when parental permission is not feasible or appropriate	May raise ethical and legal concerns regarding who qualifies as a proxy and their decision-making power. Some IRB/ethics committees allow proxy permission instead of parental permission but still prioritize the protection of minors by requiring a responsible adult to assist in decision-making
Informed Assent + Waiver of Parental permission	The minor agrees to participate (assent), and parental permission is waived by the ethics board when the research poses minimal or less than minimal risk and requiring parental permission may increase risk or harm to the minor. In some studies, additional supportive processes may be implemented—such as involving a minor advocate to help the minor make an informed decision about participation	Empowers minors, respects their autonomy, facilitates participation in sensitive research, and enhances confidentiality	Requires institution oversight (IRB/ethics committee approval); may be contested for legal or ethical reasons. Typically allowed for low-risk studies or case-by-case in sensitive contexts (e.g., sexual identity, family violence)
Emancipated or Mature Minor Consent	In specific legal or ethical contexts, minors who are legally emancipated or deemed mature (e.g., married, parenting, homeless, or financially independent) may consent to participate in research without parental permission	Respects the legal and lived autonomy of minors who function independently; reduces barriers for vulnerable populations to participate in research that may benefit them	Legal definitions vary by country or jurisdiction; requires careful documentation and justification; may still need ethics committee oversight

In practice, researchers were often concerned about how to truly preserve the rights of MSM minors while respecting their autonomy. Overly strict consent procedures, such as requiring parental permission, could have negatively affected participation and eroded trust among young MSM. This included cases where participants lived in unsupportive or violent households, did not wish to disclose their sexual orientation, or believed they had the capacity to make their own decisions. In some cases, participants distrusted their family members, making parental permission a significant barrier. When such procedures were applied without consideration for participants’ specific contexts, they hindered efforts to foster youth autonomy. In addition, lengthy procedures, complex language, or culturally insensitive materials alienated young MSM from the research process. These barriers made research feel inaccessible or difficult to understand, thereby undermining trust and reducing equitable youth participation. These challenges highlighted the need for ethically appropriate and context-sensitive approaches that both protected the rights of MSM minors and addressed Institutional Review Boards’ (IRBs) concerns about risk and participant safety [37].

The aim of this review article is to examine the consent/assent processes used when conducting HIV research with MSM under age 18 years.

## Methods

The protocol for this study was registered with the PROSPERO international prospective register of systematic reviews (registration number: CRD420251067134). This review article is carried out according to the five steps to conducting a systematic review outlined by Kahn et al. 2003 to ensure the review process is executed properly and systematically, as well as meets the review objectives [43].

- Step 1: Framing questions for a review
- Step 2: Identifying relevant work
- Step 3: Assessing the quality of studies
- Step 4: Summarizing the evidence
- Step 5: Interpreting the findings

### Framing questions for a review

This review explores the strategies and consent/assent processes used in HIV research involving MSM youths under 18. The research question was framed to address a critical gap in the literature regarding how researchers navigate legal, ethical, and practical challenges in obtaining consent for this population. Specifically, this review examines the question: ‘What strategies and considerations do researchers employ in the consent process when conducting HIV studies involving MSM minors?

### Identifying relevant work

We conducted a comprehensive literature search across three databases (PubMed, Embase, and Scopus), covering studies published between 2010 and 2023. A combination of keywords [44–46] and Medical Subject Headings (MeSH) terms was used to capture studies at the intersection of HIV, MSM, youth, and ethical processes. Specifically, the search included terms related to HIV (“HIV”, “Acquired Immunodeficiency Syndrome”, “Human Immunodeficiency Virus”), population terms (“Men who have sex with men”, “Bisexual men”, “Gay”), age-related terms (“Adolescent”, “Youth”, “Minor”, “Teenager”, “Under 18”), and ethical terms (“Consent”, “Assent”). An example of a complete search string used in PubMed was:

(((((((((AIDS) OR (“acquired immunodeficiency syndrome”)) OR (HIV)) OR (“human immunodeficiency virus”)) AND ((((“men who have sex with men”) OR (“bisexual men”)) OR (gay)) OR (“MSM”))) AND ((((((adolescent) OR (youth)) OR (minor)) OR (teenager)) OR (“Under eighteen”)) OR (“Under 18”))) AND ((consent) OR (assent)) AND ((humans[Filter]) AND (english[Filter]) AND (2010:2023[pdat]))) NOT (review)) NOT (“systematic review”)) NOT (“meta-analysis”).

The year 2010 was chosen as the starting point because it represented a transitional period in the field with increasing focus on biomedical prevention options and widespread availability of effective HIV treatment globally, allowing for an examination of research conducted over the past decade and a half while capturing the evolution of consent processes and ethical considerations leading into the present era. This systematic review focuses exclusively on English-language publications concerning human subjects, while excluding literature reviews and protocol papers. To facilitate this process, the Covidence program was employed to screen for duplicate entries. Initially, one researcher reviewed the titles and abstracts to identify relevant studies. Following PRISMA 2020 guidance [47], which permits single-reviewer screening with secondary verification, full-text screening was primarily conducted by a single reviewer (WW). To ensure accuracy and consistency, a second reviewer (TG) independently assessed a random 10% of the articles. In cases where multiple articles were available for the same study, WW and TG jointly selected the report that contained the most detailed information on consent and assent procedures. All steps in the screening process were conducted using the Covidence program [48].

According to the search results obtained from the three databases using the aforementioned search queries, PubMed generated 265 articles, while Embase and Scopus produced 64 and 74 articles respectively, additional with 7 references searched for a total of 410 articles.

### Assessing the quality of studies

The researcher assessed the quality of studies using the Mixed Methods Appraisal Tool (MMAT) checklist, a tool specifically designed to evaluate the methodological quality of studies across qualitative, quantitative, and mixed-methods designs. This checklist played a crucial role in examining the rigor, clarity, and potential biases in each study, ensuring the reliability and validity of the review’s findings. The MMAT was particularly suitable for this systematic review, as it provides a comprehensive evaluation of different study methodologies through five key questions. The first author (WW) scored all papers using Microsoft Excel. The senior author (TG) checked 10% of these for accuracy. Based on the MMAT score of 0–5, studies were categorized as follows: those with a score of 0–2 were rated as low quality, studies scoring 3–4 were classified as moderate quality, and studies with a score of 5 were considered to be of high quality [49–51].

### Summarizing the evidence

Two researchers (WW and TG) developed a table to extract key information on the consent process in HIV-related research involving MSM youths under 18. The data extraction was organized into two separate tables. The first table captures study characteristics, including research aims and objectives, study design, inclusion criteria, MSM definition, recruitment venues, incentives, and cohort retention strategies. The second table focuses on participant age, consent/assent processes, parental permission waivers, justifications for waiver requests, guardian or proxy involvement, and additional protection measures for participants. (see “Results” section).

### Interpreting the findings

The purpose of this review article was to provide an overview of studies related to the consent process in HIV research involving young men who have sex with men (MSM) under the age of 18. The results included a summary of studies conducted over the past 15 years, detailing their methodologies and the consent processes utilized. Following, we highlight the importance of the consent process and identify key associated challenges.

## Results

**Fig. 1:**
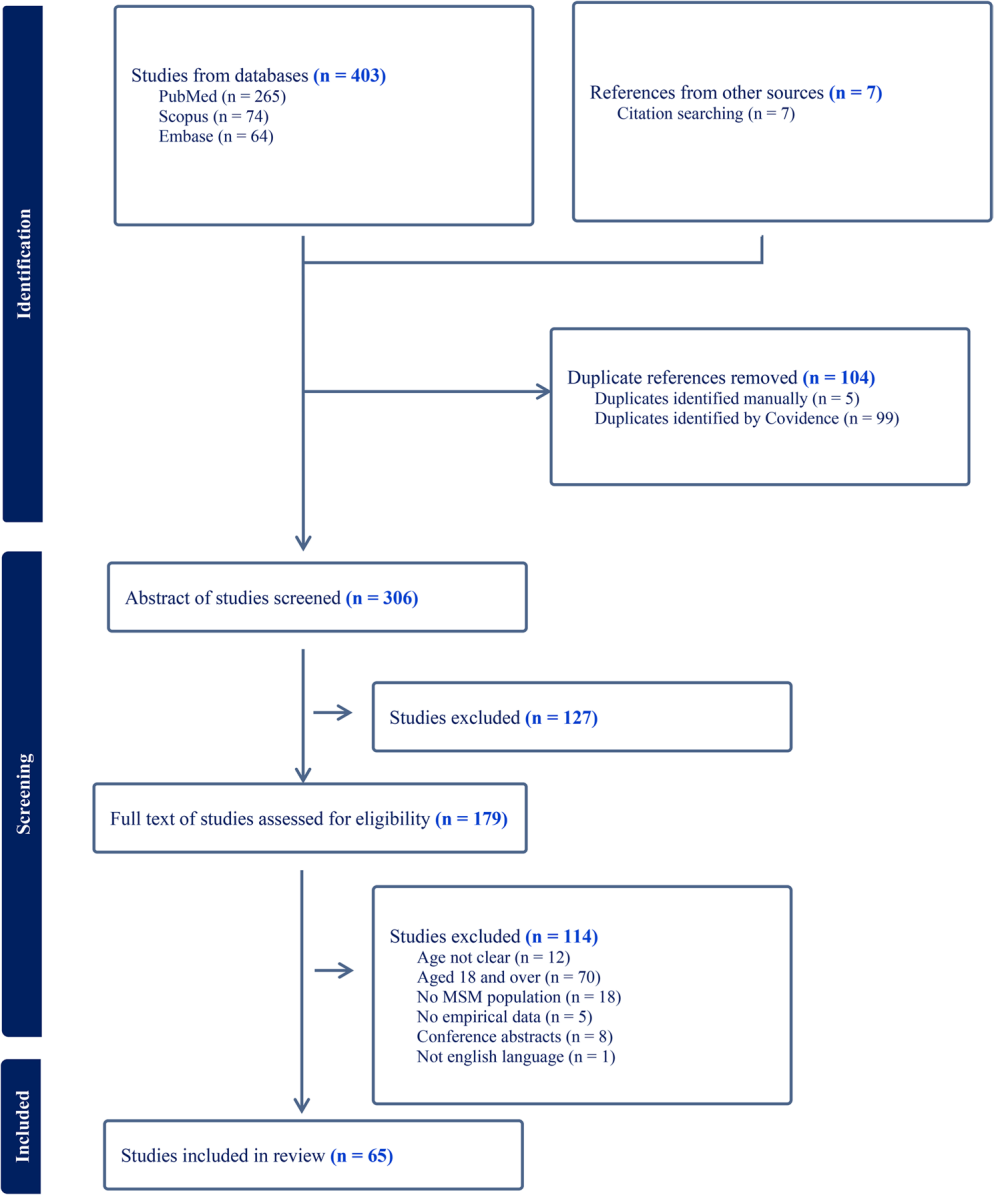
Prisma flow diagram

Four hundred and ten articles were returned from a search of 3 databases and references from other sources, after screening, 179 articles were identified that potentially met the selection criteria. After a full-text screening review, 65 of these articles were found to meet study inclusion. Below the results are presented in 3 topics as follows: (1) study characteristics; (2) study design and methodology; and (3) the consent/assent processes (Fig. 1).

### Characteristics of studies

**Fig. 2:**
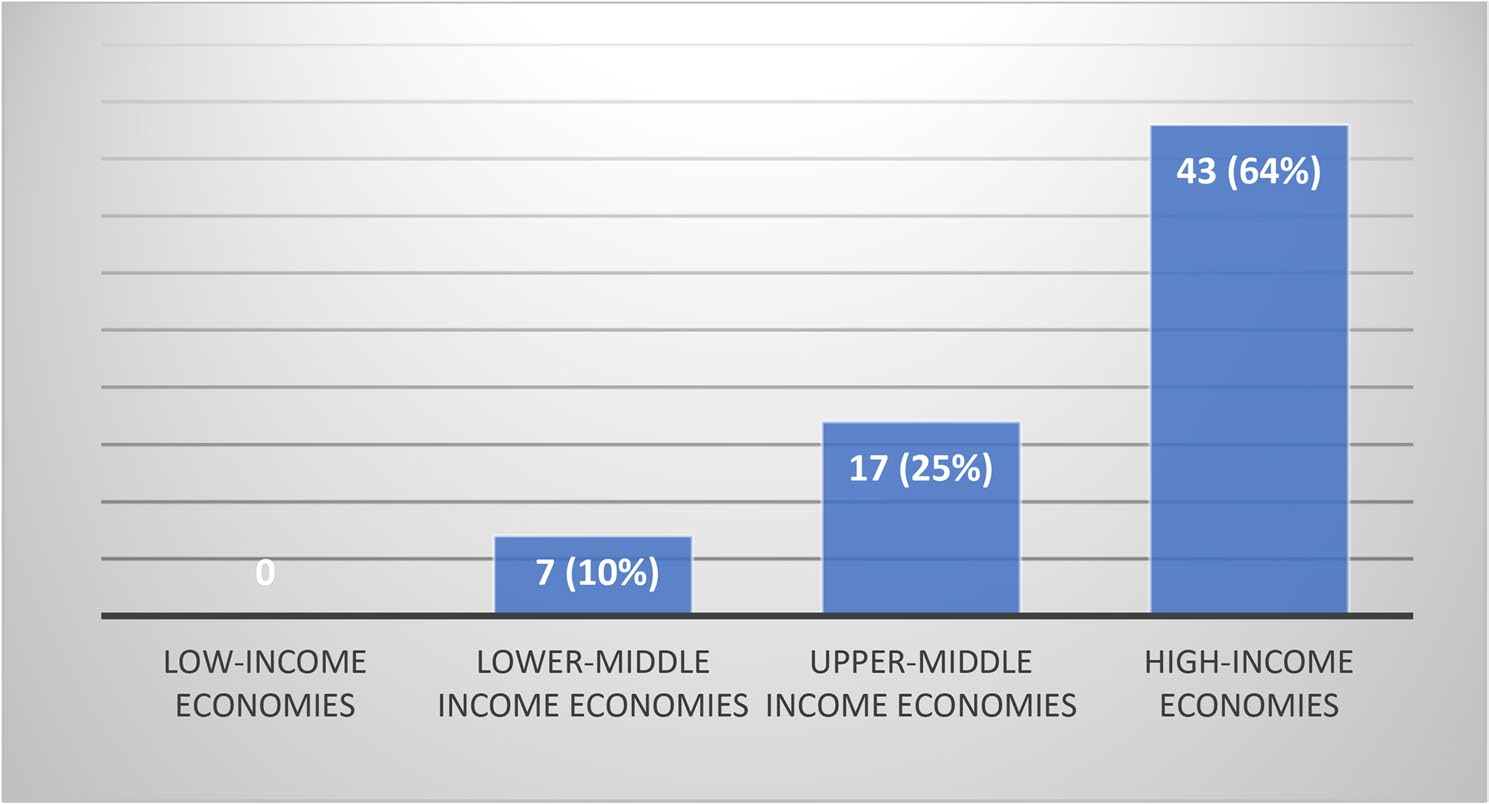
World Bank countries distribution of included studies

According to the World Bank’s [117] income level, more than half (*n* = 42, 64%) of the identified studies among MSM under the age of 18 took place in high-income countries [52–58, 60–63, 65–67, 70, 72–74, 77, 78, 80–82, 84, 88, 89, 91–95, 97–105, 108, 113]. Most of the studies were conducted in the United States, where 28 articles (43%) were published [52, 54, 56–58, 62, 63, 65–67, 70, 77, 80–82, 84, 89, 91, 92, 94, 95, 98, 99, 101–103, 105, 113]. The United Kingdom followed with 4 articles (6%), while Canada contributed 3 articles (4%) [61, 73, 93]. Additionally, there were 2 articles each from Australia (3%) [72, 97] and France (3%) [69, 85], and 1 article each from the Netherlands (1%) [55], New Zealand (1%) [53], and Switzerland (1%) [60]. Among upper-middle-income economies (17 articles, 26%), China had the highest number of studies, with 13 articles (20%) [59, 64, 75, 79, 85, 87, 96, 106, 109, 111, 112, 114, 115], followed by Brazil with 2 articles (3%) [107, 116], and Thailand (1%) [69] and South Africa (1%) [76] each with 1 article. In lower-middle-income economies, there were a total of 6 articles (9%), three of which were from India (4%) [71, 83, 86], while Nigeria (1%) [68], Pakistan (1%) [110], and Vietnam (1%) [90] each had 1 article. No studies on HIV involving young men who have sex with men (YMSM) under the age of 18 were found from low-income countries (See Fig. 2).

In this review, the term MSM (men who have sex with men) is generally defined as gay or bisexual men, or more broadly, as men who engage in sexual activities with other men. However, the operational definition of “sex” varied across studies. Some studies defined sex strictly as anal sex only (14 articles) [63, 66, 70, 75, 77, 79, 81, 85, 87, 104, 106, 108, 111, 112], while others included both anal and oral sex (6 articles) [59, 64, 71, 83, 86, 96].

Most of the studies used a broad definition, categorizing men simply as male without providing a specific definition (35 articles) [52–58, 60, 65, 68, 71, 72, 74, 75, 78, 81, 83, 84, 86, 88–90, 93–95, 97–100, 102, 107, 109, 112, 115, 116]. In contrast, 21 articles defined men as those assigned male at birth or as cisgender males [59, 62–64, 66, 67, 70, 77, 79, 80, 82, 85, 87, 92, 96, 101, 105, 106, 111, 113, 114]. Four studies included a wider definition that encompassed trans men or trans women [61, 104, 108, 110]. Five articles provided no clear information about the definition of men or males, only referring to “LGBTQ", "LGBT+", "LGBQA", "gay men", or "sexual minorities" [69, 73, 76, 91, 103] (See Table 2).

**Table 2:** Characteristics of the included studies (*N* = 65)

No.	Year	Authors	Country	Objectives^a^	Study design and inclusion criteria	MSM definition^b^	Recruitment Venue	Incentives	Cohort retention^c^	MMAT score
1	2015	Alexander et al. [52]	USA	To explore the process of adolescent decision-making on participation in an HIV vaccine clinical trial	Design: Qualitative Participants: *N* = 33 (19 MSM), aged 16–19 years, HIV-negative Description: Participants underwent a simulated HIV vaccine trial consent process and subsequently completed in-person semi-structured interviews Setting: In-person (across four urban U.S. sites)	1) Male 2) Sexually active MSM who had sex with males	1) Community-based organizations (CBOs) 2) Health clinics 3) Youth groups 4) Community events	No information provided	N/A	5/5
2	2010	Azariah et al. [53]	New Zealand	To collect data on STI and HIV diagnoses in a sample of MSM who visited the Auckland Sexual Health Service, as well as to investigate possible risk factors	Design: Quantitative (observational cross-sectional study) Participants: *N* = 87 MSM, aged 15–66 years Description: Clinic-based questionnaire (participant or clinician completed); onsite HIV/STI testing Setting: In-person (clinics)	1) Male 2) Ever had sex with a man	Auckland Sexual Health Service (ASHS)	No information provided	N/A	3/5
3	2016	Bares et al. [54]	USA	To expand Testing and Linkage to Care (X-TLC) aimed to expand routine HIV testing programs for disproportionately affected populations on the South Side of Chicago	Design: Quantitative (quasi-experimental) Participants: *N* = 67,153 minority men and women, MSM, aged 16–78 years Description: HIV testing & linkage-to-care, routine and point-of-care HIV testing comparison Setting: In-person (clinics)	Men who have sex with men	Recruitment involved routine, opt-in testing in high-prevalence communities at clinics, hospitals, and EDs.	No information provided	N/A	2/5
4	2017	Bartelsman et al. [55]	Netherlands	To evaluate whether the HTW (HIV Testing Week 2015) was an effective strategy for increasing HIV testing and detecting new infections among the targeted risk groups	Design: Quantitative (quasi-experimental) Participants: *N* = 1,231 high-risk groups (304 MSM), aged 16–81 years Description: Self-administered questionnaire and free HIV rapid/self-testing Setting: In-person testing at hospitals, GP clinics, STI clinic, outreach sites; home-based via online platform	Men who have sex with men	Recruitment took place at hospitals, GP clinics, STI clinics, outreach locations, community sites, and via home-based testing.	No information provided	N/A	2/5
5	2010	Begier et al. [56]	USA	To anonymously assess HIV prevalence among individuals entering New York City jails and approximate the percentage of cases that were previously undiagnosed	Design: Quantitative (observational cross-sectional study) Participants: 6,411 prisoners (47 MSM), aged 16 years and above Description: Information was collected through a standardized medical intake questionnaire by health care workers Setting: In-person (Jail)	Men who have sex with men	Jail	No information provided	N/A	3/5
6	2018	Bhatia et al. [57]	USA	To describe the outcomes of this implementation approach in the context of a PrEP continuum that has been developed	Design: Quantitative (observational longitudinal cohort study) Participants: *N* = 137 walk-in patients from STI clinics (126 MSM), aged 12 years and older, all HIV-negative Description: Clinical data extracted from STI clinic records and PrEP partner sites (no self-administered questionnaire used) Setting: In-person (public STI clinics; follow-up conducted by partner PrEP clinics)	Men who have sex with men	Chicago Department of Public Health (CDPH) STI Clinics.	No information provided	N/A	2/5
7	2018	Bonett et al. [58]	USA	To describe the process of constructing paradata measures based on the intervention’s theoretical framework, as well as to investigate whether participants’ sociodemographic traits are related to their overall and feature-specific use and engagement with the intervention	Design: Quantitative (Experimental) Participants: 130 MSM, aged 15–24) years Description: Online intervention to promote HIV testing Setting: Online	Men who have sex with men	1) Community-based organizations 2) Social media and dating apps (e.g., Grindr, Facebook)	Electronic gift card ($20 and an additional $10 at each follow-up)	Electronic gift card ($20 and an additional $10 at each follow-up)	3/5
8	2018	Cao et al. [59]	China	1) To examine the relationship between online STI information seeking and offline STI physician visits among YMSM 2) To examine the role of social media as a source of STI information and as a method of linking care to STI healthcare providers	Design: Quantitative (observational cross-sectional study) Participants: *N* = 503 MSM, aged 16–30 years old, all had visited a physician within the past 24 months Description: Nationwide online survey Setting: Online	1) Male at birth/cisgender male 2) Had anal or oral sex with men	Online via gay apps: WeChat, Blued, Qingtong, SESH	A small phone credit reimbursement (~$7.50 USD)	N/A	3/5
9	2016	Clerc et al. [60]	Switzerland	To examine HCV awareness (risk factors, disease course, and treatment) among MSM attending screening clinics and meeting places in southwest Switzerland against behavioral risk factors reported and examined HCV seroprevalence among MSM	Design: Quantitative (observational cross-sectional study) Participants: 654 MSM, aged 16–80 years Description: Self-administered questionnaire under field worker supervision, followed by on-site rapid HCV testing Setting: In-person (MSM clinics and indoor/outdoor meeting venues)	Men who have sex with men	1) Distribution from community-based organizations (CBOs) and health clinics 2) MSM meeting venues: indoor (e.g., saunas, sex clubs) and outdoor (e.g., Pride events, cruising areas)	No information provided	N/A	3/5
10	2019	Closson et al. [61]	Canada	To understand differences in HIV prevention awareness, access to health care, and service utilization between MSM, as well as factors associated with attendance in HIV leadership programming	Design: Quantitative (Observational longitudinal cohort study) Participants: *N* = 698 GBM, aged ≥ 16 years Description: Assessed real-world uptake, effectiveness, and impact of HIV leadership programs. Data were collected every 6 months via computer-assisted self-interview (CASI). Setting: In-person data collection at a study office in Vancouver’s West End, Canada	1) Men/transmen 2) Reported having sex with another man	Recruitment was conducted using Respondent-Driven Sampling (RDS) in Metro Vancouver, Canada.	Monetary incentives ($50 for each visit and additional $10 for recruitment of a new participant)	Monetary incentives ($50 for each visit and additional $10 for recruitment of a new participant)	4/5
11	2022	Cordoba et al. [62]	USA	This study aimed to examine if restrictive state policies requiring parental consent for HIV testing impact testing rates among young men who have sex with men (YMSM) in the USA	Design: Quantitative (observational cross-sectional study) Participants: *N* = 612 MSM, aged 13–17 years, HIV negative Description: Secondary data analysis from an mHealth HIV prevention trial Setting: Online	1) Male at birth 2) Self-identify as male or non-binary 3) Sexually attracted to male	1) Local outreach - Active recruitment in community organizations/events - Passive recruitment using flyers 2) Internet outreach via paid and targeted ads on social media platforms (IG, FB, Snapchat)	N/A	N/A	3/5
12	2011	Dorell et al. [63]	USA	To evaluate the associations between health care and HIV infection, as well as to identify missing HIV/STI testing opportunities	Design: Quantitative (Observational unmatched case-control study) Participants: *N* = 125 MSM, aged 16–25 years, both HIV-positive and HIV-negative Description: Self-administered computerized questionnaire Setting: In-person (clinics, campuses, clubs, malls, community events)	1) Cisgender male 2) Self-reported having anal sex with a man	1) Case recruitment: Identified through the HIV/AIDS Reporting System (HARS) 2) Control recruitment: Recruited from local venues (e.g., STD clinics, colleges, bars, malls, and community events) in Jackson, Mississippi	Electronic gift card ($25)	N/A	4/5
13	2017	Duan et al. [64]	China	To explore patterns of recreational drug use among men who have sex with men (MSM) in Shenzhen, focusing on preferred substances and their associations with HIV and syphilis infections	Design: Quantitative (observational cross-sectional study) Participants: *N* = 1935 MSM, aged 16–79 years Description: Self-administered computer-based questionnaire and laboratory testing for HIV and syphilis Setting: In-person (Shenzhen CDC and MSM community venues)	1) Male at birth 2) Having had sexual contact (oral or anal sex) with other males	1. Time-Location Sampling (TLS) – Bars, saunas 2. Respondent-Driven Sampling (RDS) – Participants recruit others 3. Snowball Sampling – Referrals through social networks	Monetary incentives ($7.2 and an additional $10 for the recruitment of a new participant)	Monetary incentives ($7.2 and an additional $10 for the recruitment of a new participant)	3/5
14	2016	Fisher et al. [65]	USA	To examine the attitudes of sexual and gender minority youth (SGMY) toward guardian permission for a pre-exposure prophylaxis (PrEP) adherence trial and their preparedness to provide informed	Design: Mixed methods (primarily qualitative with supportive quantitative survey items) Participants: *N* = 60 (self-identified sexually active SGMY), aged 14–17, HIV negative Description: Online questionnaire and asynchronous online focus group discussions (FGDs) Setting: Online	Men who have a sexual experience or romantic interest in the male partners	Paid Facebook advertisements targeted at youth whose profiles indicated romantic interest in same gender persons	No	N/A	4/5
15	2018	Fisher et al. [66]	USA	To investigate adolescent men who have sex with men (AMSM) attitudes toward participating in oral/injectable PrEP RCTs in order to influence future study protections of youth’s rights and welfare	Design: Quantitative (observational cross-sectional study) Participants: *N* = 198 MSM, aged 14–17 years, HIV-negative Description: Self-administered online questionnaire Setting: Online	1) Cisgender male 2) Being attracted to males 3) Reporting at least one lifetime male anal sex partner	Using Facebook advertisements	Electronic gift card ($30)	N/A	3/5
16	2020	Fisher et al. [67]	USA	To give experimental information on AMSM’s competence to consent to a randomized clinical trial assessing the safety and efficacy of oral and injectable PrEP and to inform IRB decisions on waiver of guardian permission	Design: Quantitative (observational cross-sectional study) Participants: *N* = 214 MSM, aged 14–19 years, HIV-nagative Description: self-administrated online questionnaire Setting: Online	1) Assigned male at birth 2) Reported attraction to or sexual experience with male partners	Recruited from online social media advertisements on Facebook and Instagram	Electronic gift card ($30)	N/A	3/5
17	2022	Folayan et al. [68]	Nigeria	To enhance population health by identifying effective ways to strengthen enablers and reduce obstacles to healthcare access	Design: Quantitative (observational cross-sectional study) Participants: *N* = 2119 (1,272 MSM), aged 13–72 years Description: Self-administered online questionnaire across 9 high HIV-prevalence states in Nigeria Setting: Online	Men who have sex with other men	1) Snowballing 2) Crowdsourcing by placing calls through to community members and making contact using social media platforms like Facebook, WhatsApp, and email to reach eligible participants 3) Through the National Umbrella Network for the target populations	No information provided	N/A	3/5
18	2021	Fongkaew et al. [69]	Thailand	To garner evidence, especially young people’s perspectives, to support tailored HIV preventive interventions	Design: Qualitative Participants: *N* = 25 (20 MSM, 5 TGW), aged 16–20 years Description: Four Focus Group Discussions (FGDs), segmented by age Setting: In-person (three semi-urban provinces in eastern Thailand)	No information provided (use “gay men”)	Recruited from high schools and universities in three semi-urban provinces by contacting teachers known to be LGBT allies and student clubs involving LGBT members	$10	N/A	5/5
19	2018	Frye et al. [70]	USA	To report the findings of a preliminary research phase of a bigger study aimed at informing the development of a communication technology-based intervention to increase consistent HIV testing among this age group	Design: Qualitative Participants: *N* = 30 (28 MSM), aged 16–29 years, HIV-negative or unknown status Description: In-depth interviews (IDI) Setting: In-person (New York City metropolitan area)	1) Male at birth 2) Report insertive or receptive anal intercourse with a man or transgender woman	1) Participants were recruited via platforms like Facebook and Craigslist 2) Face-to-face recruitment took place at community events like kiki balls and The Piers in NYC	Electronic gift card ($30)	N/A	5/5
20	2014	Godbole et al. [71]	India	To identify the prevalence and predictors of bisexual behavior among MSM attending TI sites, as well as the risk factors for HIV infection among MSM	Design: Quantitative (observational cross-sectional study) Participants: *N* = 4,682 MSM, aged 18–49 years (propose design 15 years) Description: Participants completed a one-page, 11-item questionnaire administered by trained counsellors and provided capillary blood samples for anonymous HIV testing Setting: In-person (19 drop-in centers across four Indian states)	1) Male 2) Had anal and/or oral sex with another male	Recruitment was conducted at 19 sites across four Indian states: Goa, Maharashtra, Gujarat, and Madhya Pradesh.	No information provided	N/A	3/5
21	2022	Gosset et al. [72]	France	To assess the proportion and the characteristics of the participants who distributed HSTs, as well as the characteristics of people who received HSTs	Design: Quantitative (observational cross-sectional study) Participants: *N* = 283 (267 MSM); aged ≥ 15 years; 86 were HIV-positive Description: Participants were recruited from a sexual health center and asked to distribute HIV self-tests to people in their networks Setting: In-person (Sexual Health Center, Paris, France)	Men who have sex with other men	LGBT + community-based a sexual health center	No information provided	N/A	3/5
22	2015	Hadland et al. [73]	Canada	To investigate the relationship between childhood maltreatment and the risk of suicide attempts among street-involved youth	Design: Quantitative (observational longitudinal cohort study) Participants: *N* = 660 street youth; aged 14–26 years Description: Interviewer-administered questionnaire and self-administered Childhood Trauma Questionnaire (CTQ) Setting: In-person (storefront location in downtown Vancouver, Canada)	No information provided (Use “Lesbian/gay/bisexual/transgender”)	Neighborhoods where street youth were known to congregate, with snowball sampling	No	N/A	4/5
23	2017	Hammoud et al. [74]	Australia	To report findings from an online prospective study on legal and illegal substance use among gay and bisexual men (GBM), and to assess baseline prevalence within the sample	Design: Quantitative (observational longitudinal cohort study) Participants: *N* = 2,250 MSM; 16–81 years Description: Nationwide sample (Australia); self-administered online questionnaire Setting: Online	1) Male 2) Identifying as a gay/homosexual or bisexual man or 3) Had sex with at least one man	The Flux study website was the main recruitment platform. Most participants joined via targeted Facebook ads.	No information provided	No	3/5
24	2018	He et al. [75]	China	To investigate factors linked to drug use and HIV infection among MSM in Hangzhou, China, with a focus on determinants associated with emerging substances like methamphetamine, ketamine, ecstasy, and rush poppers	Design: Quantitative (observational cross-sectional study) Participants: *N* = 555 MSM;; aged ≥ 16 years; HIV-negative or unknown status at baseline Description: Face-to-face questionnaire interviews followed by on-site HIV testing Setting: In-person (community-based in Hangzhou, China)	1) Male 2) Had anal sex with men	Snowball sampling	Monetary incentives ($30)	N/A	3/5
25	2017	Hogg et al. [76]	South Africa	To examine adolescents’ knowledge regarding the origin of HIV? AIDS and correlates of beliefs surrounding conspiracy in Soweto, South Africa	Design: Quantitative (observational cross-sectional study) Participants: *N* = 830 adolescents; 14–19 years; male and female Description: Interviewer-administered structured questionnaire via iPad or desktop computer Setting: In-person (Kganya Motsha Adolescent Centre and Perinatal HIV Research Unit, Soweto, South Africa)	No information provided (Use “No: Heterosexual”)	1) Kganya Motsha Adolescent Centre 2) the Perinatal HIV research Unit’s Bosha Bophelo Adolescent Health study (BBAHS)	Participants were given an honorarium of 50 ZAR ($2.67) for transportation and associated participation costs	N/A	3/5
26	2017	Hosek et al. [77]	USA	To investigate the safety and adherence to PrEP, and changes in sexual risk behavior among adolescent men who have sex with men (MSM)	Design: Quantitative (Experimental; open-label phase 2 study) Participants: *N* = 78 MSM; aged 15–17 years, HIV-negative at baseline Description: Self-administered computerized questionnaires, clinical STI testing, and biological sampling (e.g., dried blood spots for drug levels) Setting: In-person (adolescent medicine clinics in 6 US cities)	1) Male at birth 2) Anal intercourse with a male partner	1) Online social media advertisements 2) Distribution from community-based organizations (CBOs)	Mobile phone top-up ($50-$75)	N/A	3/5
27	2023	Huang et al. [78]	Australia	This study evaluates the proportion of attendees at a sexual health service who opted in or out of SHAVE	Design: Quantitative (Observation longitudinal cohort study) Participants: *N* = 18,582 (7,611 MSM); aged ≥ 16 years old Description: On-site computer-assisted self-interview (CASI), completed digitally by clients while physically present at the Melbourne Sexual Health Centre, prior to seeing a clinician. Setting: In-person (Melbourne Sexual Health Centre (MSHC), Australia)	1) Men who have sex with men 2) Men who have sex with men and women	Melbourne Sexual Health Centre (MSHC) between March 2021 and June 2022	No information provided	No	3/5
28	2019	Jin et al. [79]	China	To determine the feasibility of the Easy Test in increasing access and uptake of HIV testing and treatment services among MSM, and to identify demographic and behavioral predictors of program uptake in order to guide future implementation	Design: Quantitative (observational cross-sectional study) Participants: *N* = 879 MSM; aged ≥ 16 years old, HIV-negative or unknown status Description: Self-administered online questionnaire and home-delivered HIV self-testing kits with image upload for result confirmation Setting: Online	1) Male at birth 2) Had engaged in anal sex with a man	Online Social Media Advertisements (WeChat, QQ, and Blued)	Free test kit	N/A	3/5
29	2023	Kaufman et al. [80]	USA	This study is a usability test of a mobile app designed for the mentors of African American YMSM to increase mentors’ knowledge of and confidence in talking about HIV prevention and related topics with mentees	Design: Mixed methods Participants: *N* = 16 YMSM; aged 15–24 years Description: Pre- and post- surveys to assess changes in HIV-related knowledge, confidence, and mentoring practices. After using the UrbanMentorHub app, participants also joined FGD to share feedback on app usability, mentoring experience, and suggested improvements. Setting: In-person (Baltimore and Philadelphia), online (due to COVID-19)	1) Cisgender male 2) Had sexual interest in males or reported having sex with men	1) Community events 2) Posted on social media sites 3) Advertised on radio shows, newspapers, and gay dating apps 4) Respondent-driven sampling (RDS)	$50	N/A	4/5
30	2022	Knopf et al. [81]	USA	To explore how factors such as consent requirements, gender, sexual orientation, and characteristics of clinical trials influence minors’ willingness to participate in biomedical HIV prevention studies	Design: Quantitative (quasi-experimental) Participants: *N* = 129 LGBTQ; aged 14–17 years old; HIV-negative or unknown status Description: Participants completed simulated consent procedures and self-administered surveys using Qualtrics Setting: In-person (clinical and community facilities)	1) Male 2) Have anal sex with another man	1) Research locations: Baltimore (MD), Chicago (IL), Denver (CO), and Tampa (FL) 2) Recruitment tools: Flyers displayed in clinics and handed out at community events (e.g., Pride), along with outreach via social media platforms 3) In-person engagement: Participant recruitment conducted directly at youth health centers and local community-based organizations	$50	N/A	3/5
31	2017	Knopf et al. [82]	USA	To investigate autonomous consent and study experiences among minor and adult Project PrEPare participants, a Phase II safety study of PrEP for HIV prevention. (Project PrEPare was the first biomedical HIV prevention study in the United States to allow minors to consent autonomously for enrollment if such consent was consistent with local statutes)	Design: Mixed methods Participants: *N* = 58 MSM; aged 15–22 years Description: Self-administered online questionnaire and in-depth interviews (IDIs). The questionnaire was completed online; interviews were conducted via phone or videoconference. Setting: Online	1) Male at birth/cisgender male 2) Men who engage in sexual acts with other men	Research staff from the 10 participating Adolescent Medicine Trials Network (ATN) centers contacted eligible participants, all of whom had previously taken part in Project PrEPare, a Phase II HIV prevention study.	No information provided	N/A	4/5
32	2020	Kumar et al. [83]	India	To compare the factors associated with HIV infection among multi-risk MSM who use any substance to those who do not use any substance	Design: Quantitative (observational cross-sectional study) Participants: *N* = 23,081 MSM; aged 16–80 years Description: Data were collected through computer-assisted personal interviews (CAPI), and blood samples were obtained via dried blood spot (DBS) for HIV testing. Setting: In-person (community-based across 108 districts in India)	1) Male 2) Had anal, or oral sex with a male/hijra partner	4,067 hotspots in 108 districts across India	No information provided	N/A	3/5
33	2014	Lally et al. [84]	USA	1) To assess the effectiveness of supplementary brochures using persuasive content to enhance understanding of HIV vaccine trials, especially addressing themes related to preventive misconception 2) To examine how numeracy, health literacy, and impulsivity in decision-making relate to participants’ comprehension of key elements in an HIV vaccine trial	Design: Mixed methods Participants: *N* = 120 women and men who were sexually active with men; aged 16–19 years Description: Participants completed questionnaires and were randomized to receive no brochure, a one-sided, or two-sided brochure about HIV vaccine trials. A subset also joined follow-up interviews. Setting: In-person (four Adolescent Trials Network (ATN) sites in the U.S.)	Men who were sexually active with men	Four Adolescent Trials Network (ATN) sites	No information provided	N/A	4/5
34	2017	Li et al. [85]	China	To investigate whether peer norms and self-efficacy act as mediators in the observed relationship	Design: Quantitative (observational cross-sectional study) Participants: *N* = 1,042 MSM; aged ≥ 16 years Description: A nationwide, self-administered online questionnaire assessing community engagement, condom use peer norms, self-efficacy, and condom use behaviors using validated instruments Setting: Online	1) Male at birth 2) Having had anal sex with a man at least once during their lifetime	1) A popular online social networking platform: Danlan.org 2) Weibo and WeChat	No information provided	N/A	3/5
35	2015	Mahapatra et al. [86]	India	To produce scientific insights by analyzing socio-demographic and behavioral factors linked to HIV risk among MSM in West Bengal, based on HSS data	Design: Quantitative (observational cross-sectional study) Participants: *N* = 1,237 MSM; aged 15–49 years Description: Structured, interviewer-administered questionnaire assessing socio-demographic and behavioral characteristics; anonymous HIV testing was performed following national guidelines Setting: In-person (drop-in center across five districts in West Bengal, India)	1) Male 2) Had anal or oral sex with a male partner	Distribution from community-based organizations (CBOs)	No information provided	N/A	3/5
36	2018	Mao et al. [87]	China	1) To describe patterns of sex tourism among Chinese MSM identified as having elevated HIV risk 2) To identify socio-demographic and behavioral factors associated with sex tourism among MSM in China	Design: Quantitative (observational cross-sectional study) Participants: *N* = 1,189 MSM; aged ≥ 16 years Description: Self-administered online questionnaire conducted nationwide through gay-focused online platforms Setting: Online	1) Male at birth 2) Had anal sex with men	1) Danlan.org, an online gay portal 2) A gay mobile dating app BlueD 3) Weibo and WeChat	No information provided	N/A	3/5
37	2022	Marchant et al. [88]	UK	To implement an HIV testing strategy in emergency departments using existing biochemistry samples, aiming to increase testing uptake without altering routine clinical workflows	Design: Quantitative (observational cross-sectional study) Participants: *N* = 78,333 (males and females who get HIV test); aged ≥ 16 years Description: HIV testing was initially opt-in and later transitioned to an opt-out approach with notional consent, utilizing biochemistry samples from routine ED blood draws. Setting: In-person (Emergency Department of a large teaching hospital in London)	Men who have sex with men	OTP-out HIV testing program	No information provided	N/A	3/5
38	2021	Mata et al. [89]	USA	To explore the sexual learning experiences of bisexual male youth and their preferences regarding sexuality education	Design: Mixed methods Participants: *N* = 56 bi-male youth; aged 14–17 years; HIV-negative or unknown status Description: Data were collected through an online survey and semi-structured interviews exploring participants’ experiences with and preferences for sexuality education. Setting: In-person (e.g., at community-based LGBTQ + organizations) and online (via phone or video conferencing), depending on participant location and preference.	1) Male 2) Identified as bisexual, pansexual, or queer	1) In-person (e.g., at community-based organizations serving LGBTQ youth) 2) On social media (e.g., Facebook, Instagram)	$30 Amazon gift	N/A	4/5
39	2020	Michel et al. [90]	Vietnam	To identify the profiles of young people who use drugs (YPUD) and their HIV risk exposure in Vietnam’s three major cities, Haiphong, Hanoi, and Ho Chi Minh City (HCMC), to design a community-based HIV prevention intervention	Design: Quantitative (observational cross-sectional study) Participants: *N* = 584 YPUD; aged 16–24 years Description: Structured questionnaire administered by trained interviewers Setting: In-person interviews conducted in community settings	Men who have sex with men	1) Respondent-driven sampling (RDS) 2) Peer recruitment	Participants received VND 150,000 ($7.50 USD) for their participation and VND 50,000 ($2.50 USD) for each YPUD they helped recruit	All participants received VND 100,000 for coming back to get their screening results	3/5
40	2017	Mustanski et al. [91]	USA	Understanding teenagers’ perspectives on parental permission and the risks and benefits of participating in HIV research is critical to informing evidence-based IRB decisions	Design: Mixed method Participants: *N* = 74 sexual and gender minority adolescents; aged 14–17 years Description: Online focus group discussions and pre/post surveys exploring views on HIV research participation and parental permission. Setting: Online	1) Sexual minority (e.g., lesbian, gay, bisexual, queer, questioning) 2) Gender minority (e.g., transgender, nonbinary, genderqueer) were romantically interested in or had sex with cisgender male partners	Paid Facebook advertisements	a $30 gift card	N/A	4/5
41	2020	Nelson et al. [92]	USA	To assess the differences between Adolescent Sexual Minority Males (ASMM) with and without legal capacity to consent to HIV testing and treatment and to describe associations between legal capacity to consent and testing behavior; and to explore whether ASMM who have tested believe that parental/guardian consent was required	Design: Quantitative (observational cross-sectional study) Participants: *N* = 127 Adolescent Sexual Minority Males; aged 14–17 years Description: A self-administered online questionnaire collected data on demographics, sexual behavior, HIV testing history, and perceptions of legal consent and confidentiality. Setting: Online	1) Cisgender males 2) Self-identified as gay/bisexual, or reported male-male attraction or sexual contact	Online social media advertisements (Instagram, Facebook)	Electronic gift card ($15)	N/A	3/5
42	2019	Nelson et al. [93]	Canada	To examine the prevalence of HIV, STIs, and HIV/STI co-infections in a community-recruited, non-probability sample of MSM and to examine whether past histories of STIs were associated with current HIV infection	Design: Quantitative (observational cross-sectional study) Participants: *N* = 487 Black men (86 MSM); aged ≥ 16 years Description: Participants completed a self-administered, computer-assisted survey (ACASI) and provided biological specimens for HIV and STI testing. Setting: In-person (three community health centers across Toronto)	1) Male 2) Reported having sex with men	Distribution from community-based organizations (CBOs) and health clinics	No information provided	N/A	3/5
43	2017	Osorio et al. [94]	USA	To assess an opt-out inpatient HIV screening program that was provided using admission orders written by physician house staff. These data were compared to the number of HIV tests and diagnoses performed in the ED as part of physician-ordered HIV testing based on signs and symptoms	Design: Quantitative (observational cross-sectional study) Participants: *N* = 39,435 admitted patients and house staff medical; aged 13–64 years Description: Self-administered questionnaire. Participants completed surveys assessing their experiences, understanding, and attitudes toward inpatient opt-out HIV screening. Setting: In-person (teaching hospital in the United States)	Men who have sex with men	University of California, San Diego	No information provided	N/A	3/5
44	2013	Ott et al. [95]	USA	To address preventive misconception—where participants overestimate the level of protection they receive by participating in a prevention trial	Design: Qualitative Participants: *N* = 33 adolescents; aged 16–19 years; male, female and MSM; HIV-negative Description: Explored adolescents’ understanding of clinical trial concepts—such as experiment, placebo, and randomisation—and their perceptions related to preventive misconception in the context of a simulated HIV vaccine trial. Setting: In-person (four Adolescent Medicine Trials Network (ATN) sites)	1) Male 2) Reported having sex with men	1) Community agencies 2) Youth programmes 3) Clinics 4) Gay pride events	No information provided	N/A	5/5
45	2019	Pan et al. [96]	China	To explore key factors influencing HIV testing decisions among men who have sex with men (MSM) in China	Design: Mixed methods Participants: *N* = 803 MSM; aged ≥ 16 years; HIV-negative Description: A self-administered online questionnaire, including a discrete choice experiment (DCE), was used to assess HIV testing preferences. FGDs were conducted to identify key service attributes for the DCE design. Setting: Online	1) Male at birth 2) Had anal or oral sex with another man	1) Online social media advertisements 2) Distribution from community-based organizations (CBOs)	- $15 USD for FGD - Mobile phone top-up ($7.50) for pilot test survey	N/A	4/5
46	2020	Peuchant et al. [97]	France	To assess how common LGV is among French MSM using HIV PrEP who test positive for anorectal Chlamydia trachomatis	Design: Quantitative (observational cross-sectional study) Participants: *N* = 486 MSM; aged 17–69 years, tested positive for anorectal Chlamydia trachomatis and were using PreP Description: Prescribing clinicians completed a standardized questionnaire covering demographic, clinical, biological, and behavioral data Setting: In-person (30 hospitals and 10 STI screening centers throughout metropolitan France)	Men who have sex with men	30 hospitals and 10 STI screening centers throughout metropolitan France	No information provided	N/A	3/5
47	2018	Philbin et al. [98]	USA	To explore the association between incarceration and transactional sex among HIV-infected YMSM	Design: Quantitative (observational cross-sectional study) Participants: *N* = 97 MSM living with HIV; aged 18–24 years Description: Online survey collecting data on incarceration history, transactional sex, sociodemographic factors, drug use, and HIV care status. Setting: Online	1) Identified as male 2) Reported having sex with a man	Adolescent Medicine Trials Network (ATN) clinical sites	$25	N/A	3/5
48	2021	Phillips et al. [99]	USA	To assess whether the offer of a single, free HIV self-testing kit led to increased HIV diagnoses with linkage to care	Design: Quantitative (Observational cross-sectional study) Participants: *N* = 29,992 adolescents (10,111 MSM); aged ≤ 17 years Description: Pooled data from The 2017 local Youth Risk Behavior Survey (YRBS), assessing associations between HIV testing and state-level minor consent laws, with analysis stratified by sex and sexual behavior Setting: In-person (school-based survey across 25 U.S. jurisdictions, YRBS 2017)	1) Male 2) Reported sex with both males and females or same-sex sexual contact	High schools across 25 U.S. jurisdictions by departments of education or health	A free BioSURE self-testing kit	No	3/5
49	2013	Pollard et al. [100]	UK	To address this deficit by investigating the motivations and attitudes towards opt-out testing among different groups recruited from the community in a geographical area of high prevalence of HIV in the UK	Design: Qualitative Participants: *N* = 31 (15 women, 16 lesbian/gay/bisexual); aged ≥ 16 years Description: Community-based focus groups discussing opt-out HIV testing Setting: In-person (Brighton community centers)	Men who have sex with men	1) Local community and voluntary organizations 2) Advertisements in the local press 3) Word of mouth 4) Interested individuals emailed or telephoned through the researcher	£20 recompense	N/A	5/5
50	2017	Puckett et al. [101]	USA	To investigate negative urgency (the tendency to act impulsively in response to negative emotional experiences), positive urgency (the tendency to act impulsively in response to positive emotional experiences), and sensation seeking as independent moderators of the association of IH with binge drinking, drug use, and condomless anal sex	Design: Quantitative (observational longitudinal study) Participants: *N* = 450 MSM; aged 16–20 years Description: Self-administered online questionnaires conducted at four time points (baseline, 6, 12, and 18 months) Setting: Online	1) Male at birth 2) Identified as gay/bisexual or had sex with a man	1. Targeted in-person outreach at community venues and schools 2. Online recruitment via geo-social networking applications (e.g., Grindr, Jack’d) 3. Distribution through community-based organizations (CBOs) 4. Incentivized peer recruitment (i.e., snowball sampling)	Monetary incentives ($70 and an additional $45 at each follow-up)	Monetary incentives ($70 and an additional $45 at each follow-up)	3/5
51	2020	Rendina et al. [102]	USA	To extend prior research by monitoring trends in belief accuracy regarding the U = U message among sexual minority men (SMM), identifying related factors, and examining links between these beliefs and perceived HIV transmission risk during condomless anal sex (CAS) with undetectable partners	Design: Quantitative (observational cross-sectional study) Participants: *N* = 111,747 sexual minority men; aged 13–88 years Description: Online survey conducted in the U.S., with participants recruited via social media, dating apps, and sexual networking websites Setting: Online	1) Male 2) Report male-identified casual or main partners, or identify as a sexual minority	1) Targeted digital ads on social media platforms, including pop-ups and inbox messages within popular geolocation-based dating apps 2) Banner advertisements placed on web-based platforms used for sexual or dating networking	No incentive	N/A	3/5
52	2021	Rice et al. [103]	USA	To present the results of a quasi-experimental design that compares a PCA model delivered in drop-in centers with 3 study arms: (1) PCA selection based on AI; (2) PCA selection based on popularity, operationalized as Youth Experiencing Homelessness (YEH) with the highest DC; and (3) an observation-only comparison group (OBS)	Design: Quantitative (quasi-experimental, 3-arm study) Participants: *N* = 713 (Youth experiencing homelessness); aged 16–24 years Description: Online questionnaire and intervention conducted at 3 drop-in centers to compare the effectiveness of peer-led HIV prevention interventions Setting: Online and in-person (drop-in centers)	No information provided (use “LGBQ”)	Recruited from 3 different drop-in centers in LA	$60	N/A	4/5
53	2022	Rodger et al. [104]	UK	Aim to assess whether the offering single, free HIV self-testing kit led to increased HIV diagnoses with linkage to care	Design: Quantitative (observational longitudinal study) Participants: *N* = 10,111 MSM; aged ≥ 16 years Description: Online recruitment and data collection via sexual and social networking platforms; participants randomly allocated (3:2) to receive a free HIV self-test kit or no offer Setting: Online	1) Being a man (including transgender men) 2) Reported ever having anal intercourse with a man	Online through sexual and social networking site including Grindr, Hornet, Recon, Scruff and community FB also using Ads	A free BioSURE self-testing kit	No	4/5
54	2023	Schnall et al. [105]	USA	To evaluate the practicality of HIV self-testing (HIV-ST) among very young MSM and analyze variations in test uptake based on demographics and parental consent requirements	Design: Quantitative (quasi-experimental) Participants: *N* = 253 MSM; 14–21 years; HIV-negative or unknown status Description: Participants were contacted after completing a mobile HIV prevention intervention and offered a home HIV self-testing kit. Data collection was done via REDCap and video calls, including survey responses and test completion. Setting: Online (test kits mailed and procedures conducted virtually)	1) Male at birth 2) Self-identify as male 3) Report sexual interest in other men 4) Has either kissed another man or plan on having sex with a man in the next year	MyPEEPS Mobile Trial program	$40 electronic gift card	N/A	3/5
55	2019	Smith et al. [106]	China	To compare latent class analysis (LCA) results from two online samples of HIV-negative Chinese MSM, aiming to identify more generalizable class structures and evaluate how sampling methods influence the validity of findings	Design: Quantitative (observational cross-sectional study) Participants: (1) Nationwide online sample (*N* = 582 MSM, HIV-negative or untested, urban only) and (2) Guangzhou sentinel surveillance sample (*N* = 604 MSM, HIV-negative) Description: Self-reported behavioral surveys; HIV/syphilis testing in Guangzhou sample Setting: Online and in-person (clinic)	1) Male at birth 2) Reported ever having had anal intercourse with another man	1) Nationwide: Banner ads on BlueD app and danlan.org 2) Guangzhou: Banner ads on gztz.org with in-clinic HIV/STI testing	No information provided	N/A	3/5
56	2023	Soares et al. [107]	Brazil	To outline the process of PrEP uptake and identify factors associated with PrEP initiation among adolescent MSM and transgender women (TGW) in Brazil	Design: Quantitative (observational cross-sectional study) Participants: *N* = 903 adolescents (86.8% is MSM); aged 15–19 years Description: Analyzed baseline data from an observational cohort (PrEP1519) to assess factors associated with same-day oral PrEP initiation among adolescent MSM and TGW Setting: Online and in-person (three urban PrEP clinics in São Paulo, Salvador, and Belo Horizonte, Brazil)	1) Male 2) Had sexual practices with cisgender men, and/or TGW	1) Peer-educator-led outreach via schools, LGBTQIA + NGOs 2) Social media (Instagram, Facebook, WhatsApp) 3) Hookup apps (Grindr, Tinder, Badoo)	No information provided	N/A	3/5
57	2023	Sullivan et al. [108]	UK	To determine the size and characteristics of the population attending sexual health services (SHS) in England, assess their need and duration of need for PrEP, and evaluate both uptake and duration of PrEP use	Design: Quantitative (implementation trial with longitudinal follow-up) Participants: *N* = 24,268; aged ≥ 16 years; HIV-negative Description: Real-world implementation trial with data collected through electronic case report forms and national surveillance datasets (GUMCAD) Setting: In-person (sexual health clinics across England)	1) Cisgender males and transgender men and transgender women 2) Has anal intercourse with men	157 sexual health services (SHS) across England	No information provided	N/A	4/5
58	2019	Taklual et al. [109]	China	To evaluate the effect of route of transmission on the genotype distribution of HPgV, to determine the prevalence rate, and to identify the dominant genotypes among the two high-risk populations	Design: Quantitative (observational cross-sectional study) Participants: *N* = 201 (131 MSM and 70 IDUs); all co-infected with HIV-1; aged ≥ 15 years Description: Participants were randomly selected HIV-1 co-infected MSM and IDUs. Data were collected via structured face-to-face interviews using a pre-tested questionnaire, and blood samples were taken for HPgV RNA genotyping. Setting: In-person (Guangdong Dermatology Hospital, China)	Men who have sex with men	Guangdong Dermatology Hospital, China	No information provided	N/A	3/5
59	2021	Usman et al. [110]	Pakistan	To evaluate how acceptable oral PrEP is among MSM and transgender women (TW) in Pakistan	Design: Quantitative (observational cross-sectional study) Participants: 347 MSM and TGW; aged over 13 years; HIV negative Description: A structured, pre-tested online questionnaire administered among MSM and TW in Pakistan. Setting: Online	1) Men or transgender 2) Have sex with other man/TGW	1) Snowball sampling 2) Community groups on Facebook and Instagram 3) Community-based organizations (CBOs)	No information provided	N/A	3/5
60	2018	Wang et al. [111]	China	To examine social norm patterns and condom-use self-efficacy among Chinese MSM, and analyze their relationship with consistent condom use across partner types	Design: Quantitative (observational cross-sectional study) Participants: *N* = 1,597 MSM; aged ≥ 16 years Description: Description: Online questionnaire covered socio-demographics, consistent condom use, condom use social norms, and self-efficacy. Setting: Online	1) Male at birth 2) Engaged in anal sex with a man at least once during their lifetime	1) Danlan.org – the largest online platform serving the gay community in China 2) Associated digital platforms including the Blued dating app, as well as social media channels like Weibo and WeChat	No information provided	N/A	3/5
61	2018	Wu et al. [112]	China	To investigate how MSM looked at HIV and investigate the psychometric properties of the Chinese IPQ-R	Design: Quantitative (observational cross-sectional study) Participants: *N* = 225 MSM living with HIV; aged ≥ 16 years Description: Structured questionnaire administered through face-to-face interviews by trained peer interviewers Setting: In-person (community organizations)	1) Male 2) Reported having had anal sex with at least one man in the last 12 months	Recruited and distributed via outreach lists maintained by CBOs, which had prior service engagement with MSM living with HIV	Monetary incentives ($7.8)	N/A	3/5
62	2016	Ybarra et al. [113]	USA	To reflect on ethical considerations encountered during the implementation of Guy2Guy, a text-based HIV prevention and sexual health program, within an RCT involving sexual minority male adolescents	Design: Quantitative (Experimental, RCT) Participants: *N* = 302 sexual minority males; aged 14–18 years Description: Self-administered online questionnaires and follow-up surveys; intervention involved “Guy2Guy” – a text messaging–based HIV prevention and healthy sexuality program Setting: Online	1) Male at birth/cisgender male 2) Self-identified as gay, bisexual, or queer	Online social media advertisements	Electronic gift card ($45)	N/A	4/5
63	2016	Zhao et al. [114]	China	To assess how common recreational drug use is and examine links between poppers use and HIV/STI testing history, use of gay dating apps, and sexual behaviors	Design: Quantitative (observational cross-sectional study) Participants: *N* = 1,424 MSM; age ≥ 16 years Description: Data were collected via a self-administered online questionnaire covering drug use, sexual behaviors, app use, HIV/STI testing, and demographics. Setting: Online	1) Male at birth 2) Have sex with other men	Recruited via gay community websites and other online platforms.	No information provided	N/A	3/5
64	2012	Zhao et al. [115]	China	To assess the seroprevalence of HCV, HBV, and syphilis, and identify associated risk factors among HIV-1-infected individuals in Shandong, China, using data from participants tested between 2000 and 2010	Design: Quantitative (observational cross-sectional study) Participants: *N* = 2087 HIV-positive patients; aged 1–84 years Description: Trained medical staff administered a standardized questionnaire on demographics and risk behaviors, and collected blood samples for STI testing (hepatitis B, C, and syphilis) after HIV diagnosis Setting: In-person (multiple sites under the supervision of the Shandong Provincial Center for Disease Control and Prevention (CDC), China)	Men who have sex with men	HIV-positive individuals confirmed by the Shandong CDC between 2000 and 2010.	No information provided	N/A	4/5
65	2023	Zucchi et al. [116]	Brazil	To examine legal and ethical issues surrounding adolescent involvement in HIV research and how these may impact their best interests	Design: Quantitative (observational cross-sectional study) Participants: *N* = 347 MSM and TGW; aged 15–17 years Description: Analyzed ethical and legal barriers to enrolling adolescents (15–17) in a Brazilian PrEP project, using ethics reviews, court rulings, and participant data to assess impacts of parental consent requirements on inclusion of vulnerable youth. Setting: In-person (Public health services in three major Brazilian cities—São Paulo, Salvador, and Belo Horizonte)	1) Male 2) Engage in sexual activity with other males	Recruited in person through public health services in São Paulo, Salvador, and Belo Horizonte.	No information provided	N/A	5/5

^a^Study objectives are based upon the information gathered from selected studies

^b^Since the definition of MSM from selected studies may differ, the definition of MSM from each are provided

^c^Cohort retention means strategies for each selected study to retain participants for the longitudinal research

Only five studies specifically focused on minors MSM living with HIV. These were conducted in three countries: two in the United States [63, 98], two in China [112, 115], and one in France. The studies from the United States explored the relationship between healthcare access and HIV infection, including missed opportunities for HIV/STI testing [63], as well as the association between incarceration and transactional sex among HIV-positive YMSM [98]. The studies from China examined various health concerns, such as attitudes toward living with HIV [112] and the prevalence of co-infections like hepatitis B, hepatitis C, and syphilis [115]. The study in France focused on the distribution of HIV self-testing kits (HIVST) and the characteristics of both those who distributed and those who received them [72] (see Table 2).

### Design and methodology of studies

Most studies (53 articles, 82%) were quantitative, which were divided into (a) 36 (55%) observational cross-sectional studies, (b) 8 (12%) observational longitudinal studies, most of which collected data via questionnaires, (c) 8 (12%) experimental/quasi experimental studies, the majority of which were HIV testing interventions (4 articles; 6%), HIV prevention (3 articles; 4%), PrEP (1 article; 1%) and (d) one case control study (1%). Aside from these quantitative studies, five qualitative studies (7%) related to HIV testing and HIV vaccines were also conducted. Furthermore, there were seven articles (10%) on PrEP, HIV testing, and HIV prevention that used mixed methods (See Table 2).

Online recruitment was mainly employed to access MSM minors through online platforms and applications [59, 67, 77, 79, 85, 87, 92, 96, 101, 104, 111, 113, 114], as well as through recruitment hotspots and drop-in centers located in public areas, including hospitals, health centers, and prisons. According to most studies, community networks, such as community-based organizations (CBOs), play a crucial role in helping researchers connect with MSM minors [52, 58, 60, 62, 72, 77, 81, 86, 89, 93, 95, 96, 100, 101, 110]. They also contribute significantly to public relations, dissemination of information, and improving accessibility to MSM minors, who are often regarded as a hard-to-reach and underserved population.

A total of 29 studies (43%) offered compensation in various forms, including gift cards, cash, online shopping gift codes, mobile top-ups, and online/VISA e-gift cards. Some studies increased compensation over time [58, 61, 101]. To attract more participants, additional compensation was provided to those who invited others to join the project [61, 64, 90]. In a PrEP study conducted in the USA, the maximum compensation for each participant was a mobile phone top-up valued at $75 [77]. Furthermore, participants in a study examining the relationship between internalized homophobia, substance use, and condomless sex in the USA received monetary incentives that started at $70 at baseline, with an additional $45 given at each follow-up [101]. Most compensation ranged from $25 to $30 (See Table 2).

### Quality assessment

The majority of studies were rated as moderate quality (91%), with 6% rated as high quality, and no studies were classified as low quality. All qualitative studies were rated as high quality (100%), while all quantitative descriptive studies were rated as moderate quality (100%). Frequent methodological limitations contributing to the moderate rating included challenges related to sample representativeness and nonresponse bias. For mixed methods studies, all (100%) were rated as moderate quality, primarily due to limited reporting on how inconsistencies between quantitative and qualitative data were addressed. In quantitative non-randomized studies, 91% were rated as moderate, largely due to unclear reporting of blinding procedures for outcome assessors (see Table 3).

**Table 3:** Study design and quality assessment (MMAT)

Study Design	Low Quality (*n*)	Moderate Quality (*n*)	High Quality (*n*)	Total (*n*)
1. Qualitative			5	5
2. Quantitative Randomized Control (Trials)		4		4
3. Quantitative non-randomized		11	1	12
4. Quantitative Descriptive		37		37
5. Mixed Methods		7		7
Total	0	59	6	65

### Consent/assent process

In this review and section, we distinguished between assent, an ethical process specific to minors, and standard consent procedures for adults, as well as studies that applied both. We also considered cases involving parental or guardian permission or proxy decision-makers. Table 1 summarizes the definitions, strengths, and limitations of each consent and assent model. Details of how each study applied these approaches are presented in Tables 4 and 5. The findings under the Consent/Assent Process section are presented in three parts: (1) Consent processes (2) Assent processes and (3) Parental permission processes.

**Table 4:** Details of the consent/assent processes

No.	Year	Authors	Age of participants	Consent^c^	Assent^d^	Waiver of Parental permission	Reasons for waiver request	Guardian Involvement	Proxy involvementa	Additional protection activities^b^
1	2015	Alexander et al. [52]	16–19	Informed consent	No	Yes	No information provided	No	No	1. Adolescents reviewed a simulated HIV vaccine trial consent form 2. Research staff guided participants through key elements: purpose, procedures, risks, benefits, and compensation 3. Participants were encouraged to ask questions as part of the standard consent process
2	2010	Azariah et al. [53]	15–66	Verbal informed consent	No	No information provided	No information provided	No	No	No
3	2016	Bares et al. [54]	16–78	Verbal informed consent	No	No information provided	No information provided	No	No	1. The research team provided a patient education sheet explaining the rationale for HIV screening and associated risk factors 2. The sheet included explanations for both positive and negative test results 3. Contact information was included for the X-TLC program, as well as testing resources from the State of Illinois and the CDC
4	2017	Bartelsman et al. [55]	16–81	Written informed consent	No	No information provided	No information provided	No	No	No
5	2010	Begier et al. [56]	16 up	Written informed consent	No	No information provided	No information provided	No	No	No
6	2018	Bhatia et al. [57]	12 up	Consent	No	No information provided	No information provided	No	No	No
7	2018	Bonett et al. [58]	15–24	Online informed consent	No	No information provided	No information provided	No	No	No
8	2018	Cao et al. [59]	16–30	Online informed consent	No	No information provided	No information provided	No information provided	No information provided	No
9	2016	Clerc et al. [60]	16–80	Verbal informed consent	No	No information provided	No information provided	No	No	No
10	2019	Closson et al. [61]	16 up	Written informed consent	No	No information provided	No information provided	No	No	No
11	2022	Cordoba et al. [62]	13–17	Written informed assent	Yes (written informed assent)	Yes	No information provided	No	No	Some state policy explicitly included HIV testing among STI service available to minors without parental permission
12	2011	Dorell et al. [63]	16–25	Written informed consent	No	No information provided	No information provided	No	No	No
13	2017	Duan et al. [64]	16–79	Written informed consent	No	No information provided	No information provided	No	No	No
14	2016	Fisher et al. [65]	14–17	Verbal consent	No	Yes	No information provided	No	No	No
15	2018	Fisher et al. [66]	14 up	Independent consent	No	Yes	No information provided	No	No	No
16	2020	Fisher et al. [67]	14–19	Online informed consent	No	Yes	No information provided	No	No	No
17	2022	Folayan et al. [68]	13–72	Online informed consent	Yes	Yes	No information provided	No information provided	No information provided	No
18	2021	Fongkaew et al. [69]	16–20	Written informed consent	No	No (provided parental consent)	No	Yes	No	No
19	2018	Frye et al. [70]	16–29	Written informed consent	Yes (Written informed assent)	No information provided	No information provided	No	Minor advocate	1. Participants under 18 were considered mature minors and provided written informed assent 2. Before assent, each minor met with a minor advocate—a neutral staff member not involved in the study 3. The advocate’s role was to support the minor; discussions were confidential and not shared with study staff
20	2014	Godbole et al. [71]	15–49	Written informed consent	Yes (Written informed assent)	No information provided	No information provided	No	No	No
21	2022	Gosset et al. [72]	15 or older	Online informed consent	No	No information provided	No information provided	No information provided	No information provided	No
22	2015	Hadland et al. [73]	14–26	Written informed consent	No	Yes	No information provided	No information provided	No information provided	No
23	2017	Hammoud et al. [74]	16–81	Online informed consent	No	No information provided	No information provided	No	No	No
24	2018	He et al. [75]	16 up	Written informed consent	No	No information provided	No information provided	No	No	No
25	2017	Hogg et al. [76]	14–19	Online informed consent	No	No information provided	No information provided	No information provided	No information provided	No
26	2017	Hosek et al. [77]	15–17	Written informed consent	No	Yes	No information provided	No	No	No
27	2023	Huang et al. [78]	16 or older	Consent	No	No information provided	No information provided	No information provided	No information provided	No
28	2019	Jin et al. [79]	16 up	Online informed consent	No	No information provided	No information provided	No	No	No
29	2023	Kaufman et al. [80]	15–24	Written informed consent	No	No information provided	No information provided	No information provided	No information provided	No
30	2022	Knopf et al. [81]	14–17	Online informed consent	No	Yes	No	No information provided	No information provided	No
31	2017	Knopf et al. [82]	17–25	Online informed consent/Verbal informed consent	No	No information provided	No information provided	No	No	1. Used Likert-type questions to evaluate the consent process 2. Measured how informed participants felt 3. Assessed perceived support during decision-making 4. Evaluated whether participation was voluntary
32	2020	Kumar et al. [83]	15 up	Written informed consent	No	No information provided	No information provided	No	No	No
33	2014	Lally et al. [84]	16–19	Online informed consent	No information provided	Yes	This study as participation included only minimal risk	No information provided	No information provided	No
34	2017	Li et al. [85]	16 or older	Online informed consent	No	No information provided	No information provided	No information provided	No information provided	No
35	2015	Mahapatra et al. [86]	15–49	Written informed consent	Yes (Written informed assent)	No (consent from the next to kin, caretakers, or guardian on behalf of the minors)	No	Guardian	Kin, Caretakers	No
36	2018	Mao et al. [87]	16 or older	Online informed consent	No	No information provided	No information provided	No information provided	No information provided	No
37	2022	Marchant et al. [88]	18–59	Consent	No	No information provided	No information provide	No information provide	No information provide	No
38	2021	Mata et al. [89]	14–17	Online informed consent	Yes	Yes	No information provided	No information provided	No information provided	No
39	2020	Michel et al. [90]	16–24	Written informed consent	No	No information provided	No information provided	No	No	No
40	2017	Mustanski et al. [91]	14–17	Online informed consent	Yes (Online inform consent)	Yes	IRBs can approve waivers of parental permission requirements when studies are not otherwise feasible or when parental permission may not protect the adolescents. Adolescents’ perspectives represent a critical lens through which to examine these issues.	No information provided	No information provided	No
41	2020	Nelson et al. [92]	14–17	Online informed consent	No	Yes	No information provided	No	No	1. Capacity to consent assessed using four comprehension questions 2. Questions covered study procedures, risks, and benefits 3. Participants had up to three attempts to answer correctly 4. Those unable to answer all correctly were deemed ineligible
42	2019	Nelson et al. [93]	16 up	Written informed consent	No	No information provided	No information provided	No	No	No
43	2017	Osorio et al. [94]	13–64	Verbal informed consent	No	No information provided	No information provided	No	No	No
44	2013	Ott et al. [95]	16–19	Written informed consent	No	Yes	No information provided	No		No
45	2019	Pan et al. [96]	16 up	Online informed consent	No	No information provided	No information provided	No	No	No
46	2020	Peuchant et al. [97]	17–69	written informed consent	No	No information provided	No information provided	No	No	No
47	2018	Philbin et al. [98]	13–24	Written informed consent	No	No information provided	No information provided	No information provided	No information provided	No
48	2021	Phillips et al. [99]	16 or older	Online informed consent	No	No information provided	No information provided	No information provided	No information provided	No
49	2013	Pollard et al. [100]	16–65	No information provided	No	No information provided	No information provided	No information provided	No information provided	No
50	2017	Puckett et al. [101]	16–20	Informed consent	Yes (Informed assent)	Yes	No information provided	No	No	No
51	2020	Rendina et al. [102]	13 or oder	Online informed consent	Yes	Yes	No information provide	No information provide	No information provide	No
52	2021	Rice et al. [103]	16–24	Consent	No	No information provided	No information provided	No information provided	No	No
53	2022	Rodger et al. [104]	16 or older	Online informed consent	No	No information provided	No information provided	No	No	No
54	2023	Schnall et al. [105]	14–21	Online informed consent	Yes	No (parental e-consent)	No information provided	No information provided	No information provided	No
55	2019	Smith et al. [106]	16 or older	Consent	No	No information provided	No information provided	No information provided	No information provided	No
56	2023	Soares et al. [107]	15–19	Written informed consent	Yes (Written)	Yes	No information provided	No information provided	No information provided	No
57	2023	Sullivan et al. [108]	16 or older	Written informed consent	No	No information provided	No information provided	No information provided	No information provided	No
58	2019	Taklual et al. [109]	15 or older	Written informed consent	No	No information provided	No information provided	No information provided	No information provided	No
59	2021	Usman et al. [110]	18–67	Online informed consent	No	No information provided	No information provided	No	No	No
60	2018	Wang et al. [111]	16 or older	Online informed consent	No	No information provided	No information provided	No information provided	No information provided	No
61	2018	Wu et al. [112]	16 up	Written informed consent	No	No information provided	No information provided	No	No	No
62	2016	Ybarra et al. [113]	14–18	Informed consent	Yes (Informed assent)	Yes	No information provided	No	No	1. Enrollment conducted via phone to assess minors’ understanding of assent. 2. Assent form written in simple, age-appropriate language; capacity to consent assessed to ensure understanding of risks and voluntariness. 3. Waiver of parental permission obtained from both IRBs due to minimal risk and sensitivity of the study. 4. Self-safety assessment developed to help youth evaluate whether it was safe for them to participate. 5. Step-by-step privacy instructions provided (e.g., setting a phone password) to enhance participant confidentiality.
63	2016	Zhao et al. [114]	16 or older	Written informed consent	No	No information provided	No information provided	No information provided	No information provided	No
64	2012	Zhao et al. [115]	1–84	Informed consent	No	No information provided	No information provided	Questionnaires were completed by guardians for participants with developmental, physical, or cognitive limitations	No	No
65	2023	Zucchi et al. [116]	15–17	Consent	Yes	No (parental consent were adopted across sites)	No information provided	No information provided	No information provided	No

^a^Proxy involvement refers an adult person that is not a legal guardian to give permission and helps minors make decisions

^b^Additional activities are organized in selected HIV research studies to protect minors, minimize harms, prevent risk, and protect their rights

^c^Informed consent as stated in the paper

^d^Informed assent as stated in the paper

**Table 5:** Summary table of consent and assent practices (*n* = 65 studies)

Consent/Assent Practice	Number of Studies	References
Reported use of any informed consent process	64	[52–116] exclude [100]
Used online consent forms	22	[58, 67, 68, 72, 74, 76, 79, 81, 82, 84, 85, 87, 89, 91, 92, 96, 99, 102, 104, 105, 110, 111]
Did not distinguish between minors and adults in consent	51	[52–116] exclude [62, 68, 70, 71, 86, 89, 91, 100–102, 105, 107, 113, 116]
Used comprehension questions check as part of consent	1	[92]
Used participant-centered tools (e.g., safety self-assessments)	2	[82, 113]
Included pre-participation discussions	1	[54]
Reported a distinct assent process for minors	13	[62, 68, 70, 71, 86, 89, 91, 101, 102, 105, 107, 113, 116]
Used written assent forms	4	[62, 70, 71, 107]
Included minor-specific protections (e.g., simplified language, privacy tools)	3	[70, 92, 113]
Involved minor advocates	1	[70]
Reported waiver of parental permission	18	[52, 62, 65–68, 73, 77, 81, 84, 89, 91, 92, 95, 101, 102, 107, 113]
Provided justification for parental permission waiver	2	[84, 91]
Required parental permission	3	[69, 86, 116]
Mentioned guardian or kin involvement	1	[86]
Reported variation in parental permission by region	1	[116]

#### Consent processes

Out of all **6**5 studies reviewed, **6**4 reported implementing a consent process. Among these, 22 studies employed an online form of informed consent [58, 67, 68, 72, 74, 76, 79, 81, 82, 84, 85, 87, 89, 91, 92, 96, 99, 102, 104, 105, 110, 111]. In most studies, the consent process was presented collectively for all participants, with no clear distinction between adults and minors. Furthermore, a distinct procedure for obtaining assent from minors was typically not described; rather, studies generally reported that consent had been obtained from all participants.

However, one study [92] required participants to complete a comprehension questionnaire prior to enrollment. Participants had to answer all questions correctly, and those who failed to do so three times were deemed ineligible to participate. This study included a process specifically designed for minors, as all participants were under the age of 18. Two studies also introduced participant-centered tools, such as self-safety assessments, to enhance informed decision-making [82, 113]. Additionally, some studies offered pre-participation discussion sessions, allowing potential participants to reflect on the study’s details before providing consent [54] (See Tables 4 and 5).

#### Assent processes

Only thirteen studies involving minors under the age of **1**8 included an assent process [62, 68, 70, 71, 86, 89, 91, 101, 102, 105, 107, 113, 116]. Four of these used written informed assent [62, 70, 71, 107], and three studies [70, 92, 113] applied a distinct and protection process specifically designed for minors. Innovative strategies were identified in some studies to support minor participants’ autonomy and understanding. One study used simplified language and included a self-safety assessment, along with instructions on configuring phone security settings to promote participant safety and privacy [113]. In another study, minor advocates were engaged to support MSM minors under **18**, minor advocates were not part of the research team and served as intermediaries to address participants’ concerns confidentially before enrollment [70] (See Tables 4 and 5).

#### Parental permission processes

Out of 65 studies, a waiver of parental permission was explicitly reported as required in only eighteen studies [52, 62, 65–68, 73, 77, 81, 84, 89, 91, 92, 95, 101, 102, 107, 113]. Only two studies provided justifications for requesting a waiver of parental permission—one citing minimal risk [84], and the other emphasizing the protection of minors [91].

Out of the 65 studies, only three required parental permission. These include a qualitative study that developed and evaluated a participatory sexual health program tailored to the needs of YMSM in Thailand [69], and a qualitative study conducted in India that explored the experiences and challenges faced by young MSM in accessing sexual and reproductive health services. Notably, only this study [86] explicitly mentioned the involvement of kin, caretakers, or guardians acting on behalf of minors. In addition, one quantitative study [116] examined the ethical and legal challenges of providing daily oral PrEP to adolescent MSM and transgender women aged 15–19 in Brazil. This study involved both quantitative data analysis and an in-depth review of decisions made by research ethics committees across different regions. Interestingly, the requirement for parental permission varied by location—some regions mandated it, while others granted a waiver, allowing adolescents to participate independently (See Tables 4 and 5).

## Discussion

This review highlights key limitations in the ethical reporting of HIV research involving MSM minors. Most studies were conducted in high-income countries, particularly the United States, with little representation from low-income settings. This imbalance may reflect disparities in research infrastructure, legal constraints, or funding priorities, which have been similarly noted in prior reviews on adolescent research ethics [118]. In addition, sociocultural stigma [36, 119], criminalization of same-sex relationships [29], and heightened ethical and regulatory barriers to enrolling sexual minority minors may further limit research conducted in low-income settings. Notably, only three studies focused specifically on MSM minors living with HIV, despite growing evidence that this group experiences unique barriers to care, support, and participation in research [120].

The reviewed studies were predominantly quantitative and observational, with most using convenience sampling via social media or community venues. While these methods can facilitate access to hidden populations, they may underrepresent MSM minors who are most marginalized or disengaged from services [121]. A minority of studies used experimental designs or qualitative methods, and few clearly described peer referral processes or safeguards. Although 43% of studies reported offering incentives, few explained how these were delivered or ethically justified. This lack of detail raises concerns about potential undue influence, particularly among minors who may be economically or socially vulnerable. In such contexts, incentives may support recruitment but could also compromise voluntary participation if decision-making capacities are still developing [122].

The limited reporting on informed assent is particularly striking. Only thirteen studies explicitly mentioned an assent process for minors, and even among those, few tailored the process to age or developmental stage. This echoes findings by Soll et al. (2020) [118], who noted a similar lack of attention to assent procedures in adolescent health research. Despite ethical guidelines recommending that minors be given age-appropriate opportunities to provide informed agreement to participate (CIOMS, 2016) [123], our review suggests this standard is often underreported, if not entirely overlooked.

Furthermore, variability in how studies navigated parental permission, such as through formal waivers, proxy consent, or omission of detail, reflects broader inconsistencies in ethical oversight. For example, while 18 studies mentioned receiving IRB-approved waivers of parental permission, few explained the rationale or described how MSM minors were otherwise protected. These patterns may stem from ambiguous regulatory guidance, legal uncertainty surrounding minors’ autonomy, or assumptions that standard adult procedures suffice [124, 125].

Many studies applied consent procedures to minors similarly to adults, despite ethical guidelines recommending additional protections and the inclusion of assent for minors. However, only a limited number of studies provided detailed descriptions of how assent was obtained, which constrains transparency and limits opportunities for methodological learning or replication. This pattern suggests that minors’ perspectives and decision-making roles may not always be explicitly foregrounded in reporting practices. Existing literature highlights that well-designed assent processes can support trust, shared understanding between researchers and participants, and minors’ capacity to make informed decisions about research participation [126, 127]. Existing literature has suggested that assent may be appropriate for children as young as 10 years of age, depending on developmental capacity and context [128]. In digital research settings, interactive approaches such as question-and-answer formats have been proposed to enhance minors’ understanding of study procedures, risks, benefits, and their right to withdraw [45]. Ethical guidance further emphasizes the importance of age-appropriate language, sufficient time for comprehension, and context-sensitive protections when conducting research with minors [45, 129, 130]. Nevertheless, relatively few studies in this review reported implementing or describing such tailored approaches in detail [54, 70, 82, 92].

Nearly half of the studies included in this review (43%) reported providing compensation to participants, with amounts ranging from $25 to $75. Some studies also mentioned additional incentives for peer referrals. This observation raises potential ethical considerations, particularly in studies involving MSM minors. While compensation may encourage participation and help reduce dropout rates, especially among youth who are economically or socially marginalized, it may also raise concerns about undue influence, depending on the context. In HIV research, where participants may experience intersecting vulnerabilities related to stigma, economic disadvantage, and limited legal protections, the use of incentives calls for careful ethical reflection. Ensuring that participation is both voluntary and informed is essential, and compensation should not compromise participants’ ability to assess personal risks. This consideration is especially relevant for minors, whose autonomy and decision-making capacity are still developing. Some literature has noted that adolescents may perceive incentives differently depending on their individual circumstances; some may find them supportive, while others may feel uncertain or pressured [122]. In this review, few studies provided detailed descriptions of how incentives were presented within consent or assent procedures. Greater clarity in future reporting could support transparency and assist researchers and ethics committees in considering context-sensitive approaches.

Why is assent missing? While assent procedures were present in some studies, they were reported inconsistently and often lacked sufficient detail. This pattern suggests that the role of assent may be underreported or de-emphasized in published HIV research involving minors. Researchers working with vulnerable minors may encounter practical challenges when navigating ethical frameworks that vary across settings and are sometimes perceived as unclear or inconsistently applied [116]. Such variability may reflect efforts to maintain flexibility and allow for cultural and social sensitivity. At the same time, the limited reporting of assent procedures may also reflect cautious approaches to participant protection, potentially influenced by institutional or regulatory considerations. This observation raises an ethical question regarding how IRBs balance the protection of vulnerable participants with respect for minors’ emerging autonomy. This issue remains insufficiently examined in the current literature [116]. Additionally, a substantial proportion of the reviewed studies did not provide detailed descriptions of consent and assent procedures, which limits transparency and comparability across studies. This pattern may reflect assumptions that assent procedures are embedded within local ethical or cultural contexts, or that IRB approval implies adequate ethical oversight without the need for further reporting. Alternatively, authors may assume that such procedures are widely understood and therefore do not require explicit description. These possibilities highlight a gap in empirical reporting rather than definitive conclusions about intent or ethical commitment. Addressing the persistent lack of detailed reporting on assent procedures may therefore require broader, coordinated efforts, including international collaboration and shared ethical dialogue among researchers, ethics committees, and journal editors.

### Strengths and limitations

This systematic review found that one in five studies involving MSM minors reported an assent process. This reporting gap highlights the need for clearer expectations in adolescent HIV research. To promote ethical rigor and transparency, journals and ethics committees should require explicit reporting of assent and consent methods. Addressing this gap can improve protections for adolescent participants and foster shared standards across research contexts. However, this study has several limitations. The search was conducted in only three databases and focused solely on English-language studies, which may have limited the comprehensiveness of the findings and introduced language bias. In addition, the search terms focused on MSM under the age of 18 years, meaning studies that did not explicitly state the inclusion of this group may have been missed. The review also excluded grey literature and unpublished materials, including internal IRB documents, which restricts the ability to fully assess ethical practices, particularly in settings where such information is not publicly accessible. Moreover, reporting on assent procedures was often limited or inconsistent across the included studies, which may have led to an underestimation of existing ethical practices that were implemented but not described in the published articles. Finally, most of the reviewed studies were conducted in high-income countries, limiting the generalizability of the findings to lower- and middle-income settings, which were underrepresented in this review. Developing an evidence base in these settings remains a critical priority.

## Conclusion

These findings underscore significant gaps in research on HIV among MSM minors, particularly in low-income countries. Moreover, consent and assent processes were often reported with limited clarity, blurring distinctions between procedures for MSM minors and those aged 18 years and older. This lack of transparency may contribute to assumptions that identical consent procedures apply to both groups, despite important ethical differences. The limited and inconsistent reporting of assent procedures may reflect challenges related to ethical documentation, variability in legal frameworks, cultural norms surrounding minors, or differing IRB requirements across settings. As a result, assent procedures may be underreported or insufficiently described in published studies, rather than entirely absent. Given that only one in five studies involving MSM minors reported an assent process, this gap presents an opportunity for researchers, IRBs, and journal editors to strengthen transparency, promote shared ethical understanding, and support assent practices that appropriately respect both the protection and emerging autonomy of MSM minors in HIV research.

To promote ethical integrity and transparency in HIV research involving MSM minors, the following recommendations may be considered:

1. Clearly report consent and assent procedures to support transparency and shared learning. Describe how minors were informed, how consent/assent was obtained, and whether waivers or proxies were used. Detailed reporting allows future researchers to build upon ethical practices in diverse contexts.
2. Include underrepresented populations. Prioritize MSM minors living with HIV and those from under-resourced or legally restrictive environments to address research gaps.
3. Tailor assent procedures to age and cultural context. Ensure that processes reflect minors’ developmental maturity and are sensitive to local norms.
4. Use accessible, age-appropriate language. Design study materials that are clear and understandable for youth participants.
5. Provide adequate time for questions and decisions. Allow minors space to process information and ask questions before enrolling.
6. Justify the use of incentives. Clearly explain the rationale, method of delivery, and safeguards to ensure that incentives do not compromise voluntary participation.

## Data Availability

The datasets used and/or analyzed during the current study are available from the corresponding author upon reasonable request.
